# Multi-Enzyme Assembly on T4 Phage Scaffold

**DOI:** 10.3389/fbioe.2020.00571

**Published:** 2020-06-24

**Authors:** Jinny L. Liu, Daniel Zabetakis, Joyce C. Breger, George P. Anderson, Ellen R. Goldman

**Affiliations:** Center for Bio/Molecular Science and Engineering, Naval Research Laboratory, Washington, DC, United States

**Keywords:** phage scaffold, Hoc, SpyTagSpyCatcher, amylase, maltase, glucokinase

## Abstract

Over the past two decades, various scaffolds have been designed and synthesized to organize enzyme cascades spatially for enhanced enzyme activity based on the concepts of substrate channeling and enhanced stability. The most bio-compatible synthetic scaffolds known for enzyme immobilization are protein and DNA nanostructures. Herein, we examined the utility of the T4 phage capsid to serve as a naturally occurring protein scaffold for the immobilization of a three-enzyme cascade: Amylase, Maltase, and Glucokinase. Covalent constructs between each of the enzymes and the outer capsid protein Hoc were prepared through SpyTag–SpyCatcher pairing and assembled onto phage capsids *in vitro* with an estimated average of 90 copies per capsid. The capsid-immobilized Maltase has a fourfold higher initial rate relative to Maltase free in solution. Kinetic analysis also revealed that the immobilized three-enzyme cascade has an 18-fold higher converted number of NAD^+^ to NADH relative to the mixtures in solution. Our results demonstrate that the T4 phage capsid can act as a naturally occurring scaffold with substantial potential to enhance enzyme activity by spatially organizing enzymes on the capsid Hoc.

## Introduction

Catalytic properties of enzymes are greatly affected by their surrounding microenvironment, particularly enzymes retained in a small area either by limited surface or restricted volume (Kuchler et al., 2016). Specifically, high concentration of enzymes in a confined environment are more stable than those free in solution. As a result, there is a preference for a folded state over an unfolded state and their close proximity allows them to execute a series of biocatalytic events more efficiently through substrate channeling (Miles et al., 1999; Zhang, 2011). Based on these concepts, various synthetic scaffolds aimed to spatially organize enzymes were designed and used for immobilizing enzymes to enhance their activity (Conrado et al., 2008; Dueber et al., 2009; Fu et al., 2014). Artificial multi-enzyme scaffolds have been utilized both *in vivo* and *in vitro* to improve product production (Siu et al., 2015; Ellis et al., 2019). Among these synthetic scaffolds, protein arrays and DNA nanostructures are the most biocompatible and have the potential to form the basis of a powerful platform to enhance multi-enzyme catalysis for biotechnology applications (Klein et al., 2019; Lim et al., 2019). Our team is researching naturally occurring scaffolds possessing the ability to spatially organize enzymes.

One benefit that naturally occurring phage scaffolds possess is that they are monodisperse and can be produced economically from bacteria hosts. In addition, most of the icosahedral phage capsids are composed of arrays of hexamers formed by capsid proteins, which can serve as a platform for immobilizing enzymes spatially. T4 capsids are composed of the major capsid protein, gp23, and minor capsid protein, gp24, along with two accessory proteins, Hoc (highly antigenic outer capsid) and Soc (small outer capsid), and the portal protein, gp20 (Rao and Black, 2010). The capsids are homogeneous in size and structure and can only assemble inside the host bacteria with the expression of phage chaperone proteins and proteases. During the early infection of phage in bacteria, gp23 and gp24 assemble into a prohead shell wrapping around the core structure, later removed by protease. The vacant proheads allow DNA packaged inside through a packaging machinery, followed by the attachment of tail and tail fibers. Without DNA packaging, tail and tail fibers do not attach to the capsids.

The biggest hurdle for using phage scaffolds for displaying protein is that most capsids are quite rigid and can only display short peptides or few numbers of large proteins *in vivo* (Cardinale et al., 2012; Patel et al., 2017). However, T4 phage capsids allow one to overcome this difficulty by providing the needed flexibility to enable the display of large proteins through fusion with either of two outer capsid proteins, Hoc and Soc, without disrupting the capsid structure (Ren and Black, 1998). Moreover, both Hoc and Soc are dispensable for T4 phage propagation, their absence having no impact on T4 production. Soc proteins are closely associated with capsid proteins and are assembled next to each other, while Hoc is located at the center of hexamers, separated by both capsid proteins and Soc, based on a cryo-electron microscopy (EM) model (Fokine et al., 2004). Another advantage is that both Hoc and Soc fusions can be assembled onto Hoc and Soc deletion phage capsids as a scaffold either *in vitro* or *in vivo* (Rao and Black, 2010).

Our previously established work on characterization of T4 phage capsid structure using atomic force microscopy (AFM) showed that purified capsids are intact and stable (Archer and Liu, 2009; Robertson and Liu, 2012), and based on this work, we developed a new strategy to display a multi-enzyme cascade on phage capsids through SpyTag(St)/SpyCatcher(SC) pairing (Reddington and Howarth, 2015). Amylase (Aml), Maltase (Mal), and Glucokinase (GK) were selected to assemble onto phage scaffolds for catalytic analysis. These three enzymes are part of a four-enzyme biocatalytic pathway, which converts maltoheptaose into NADH and 6-phosphogluconolactone and the bio-catalytic assays for analyzing the enzyme activity have been well-established (Klein et al., 2019). The assembly of Hoc fusions onto phage scaffolds was conducted *in vitro* for better characterization. Enzyme–SC–St–Hoc fusions were purified and incubated in controlled ratios with T4 capsid. As observed in other scaffold systems, when assembled on the T4 capsid, the spatially organized assembled enzymes exhibit enhanced enzyme catalytic activity. The immobilized enzyme fusions on the phage scaffold showed enhanced biocatalytic activity for the number of NAD^+^ converted to NADH per second up to 18-fold higher than the enzyme fusions free in solution. We have successfully demonstrated T4 icosahedral phage as a naturally occurring scaffold adaptable for multi-enzyme cascade assembly.

## Materials and Methods

### Construction of Enzyme–SC Fusions and St–Hoc Plasmids

All the primer sequences used for cloning are listed in Table S1 and gene maps and sequences are described in the Supplementary Information. The enzyme–SC fusions were cloned into pET28, while St–Hoc was inserted into pACYCduet according to Anderson et al. (2010) and Goldman et al. (2017). Details are provided in the Supplementary Information.

### Preparation of Fusion Proteins

Protein was produced using a protocol similar to one that had been previously described with several modifications (Walper et al., 2014). The protocol for protein production and purification is provided in the Supplementary Information. Protein concentrations were determined using UV-Visible spectroscopy and the molar extinction coefficient predicted from the protein sequence.

### Preparation of Tailless ΔHoc T4 Phage Scaffolds

*E. coli*, CR63 (F^−^ *supD60 lamB63*), or B40I (F^−^*supD*), containing tRNA suppressor E, was used for growing del Hoc T4 phage (ΔHoc T4), a gift from Dr. Lindsay W. Black, Professor of Biochemistry Department, UMB. The phage capsid preparation was performed according to Liu et al. (2014). Details are provided in the Supplementary Information. The concentration of phage capsids was estimated based on 1 mg = 10^13^ capsids by taking into account the copy numbers of capsid proteins, Hoc, Soc, and portal proteins in one capsid (Fokine et al., 2004; Liu et al., 2014). The protein concentration was determined by the absorbance 280 nm and Bradford assay (Bio-Rad, Hercules, CA).

### Assembly of Hoc–Enzyme Fusions Onto ΔHoc T4 Phage Capsids

The three model enzyme–Hoc fusions (Aml–Hoc; Mal–Hoc; and GK–Hoc) were first mixed at various molar ratios, 1:1:1; 1:4:1; 1:16:1. Enzyme–Hoc mixtures were subsequently added to phage capsids at ratios of total enzyme to phage capsid of 60:1 or 40:1. The same amount of enzyme–Hoc fusions was also prepared without added phage capsids. The mixtures were incubated at 22°C for 2 h or overnight (16 h). The assembly buffer consisted of PBS supplemented with 10 mM MgCl_2_. To assess assembly, after incubation, the reactions were loaded onto 1% agarose and passed through 100 V for 2 h in 1 × Tris–Acetate–EDTA buffer. The scaffolds and proteins were then visualized with GelCode blue safe protein stain (Thermo Fisher Scientific).

### Measurements of Enzymatic Activity

For assessing Maltase activity, the production of 4-Nitrophenol (absorbance at 405 nm) as a function of time was recorded for 3–4 h using a Tecan Infinite M1000 plate reader immediately after the addition of the substrate, 10 mM 4-Nitrophenyl-α-D-glucopyranoside to a mixture of Maltase or Mal-Hoc fusions in 250 mM HEPES (pH 7.4) buffer. For measuring the three-enzyme cascade activity, glucose-6-phopsphate dehydrogenase was included in excess in the reaction mixture and the rate was measured as NAD^+^ conversion to NADH (absorbance at 340 nm) as a function of time after the addition of Maltoheptaose. The reaction mixture included 5 mM ATP, 1 mM NAD^+^, and 10 U of glucose-6-phosphate dehydrogenase (G8404-2KU) in 250 mM HEPES (pH 7.4). The reaction schemes are described in more detail in Figure S1. The initial rate was measured as the slope after conversion of absorbance to nM/s, and *V*_max_, *K*_cat_, and *K*_m_ were calculated using Michaelis-Menton kinetics. Measurements were performed in triplicate. Chemicals were purchased from Millipore Sigma (St. Louis, MO), unless otherwise mentioned. Error bars were standard deviation derived from either biological replicates or technical triplicates.

## Results

### Demonstration of Purified Enzyme–Hoc Assembled on Phage Capsids

**Figure 1:**
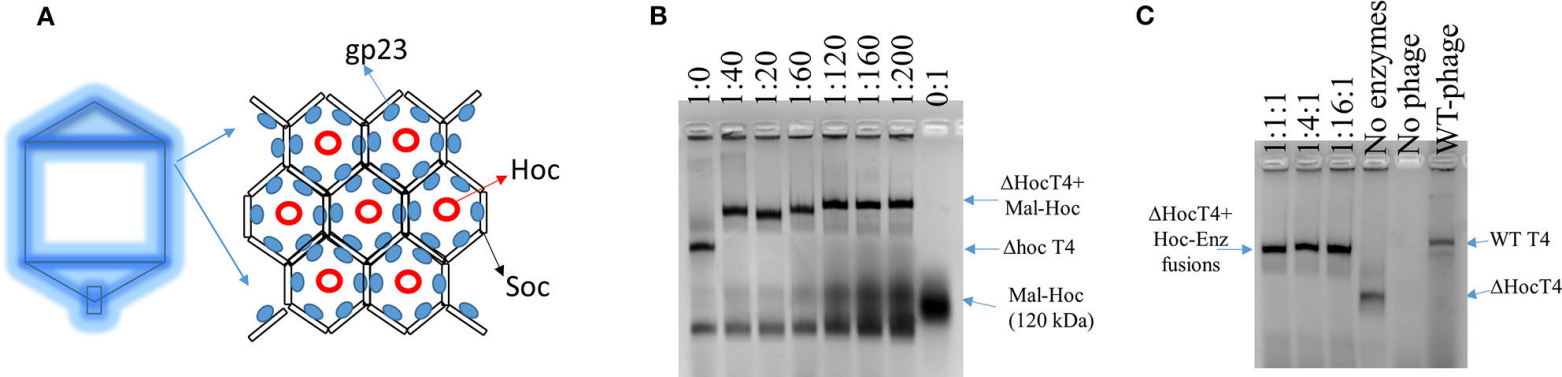
Scheme of T4 capsid hexamers and the assembly of Hoc–enzyme fusions onto ΔHoc T4 phages. **(A)** T4 capsid protein, gp23, forms hexamers and Hoc is located at the center of the hexamers. **(B)** The agarose image of mixtures of phage scaffolds and Maltase-Hoc fusions at a series of ratios. Ratios are given above each lane as phage scaffold: Mal–Hoc. **(C)** The agarose image of mixtures of the phage scaffolds and three Hoc–enzyme fusions, Aml–Hoc, Mal–Hoc, and GK–Hoc, with the total sum concentration 60 nM, which have a 60:1 ratio for the total enzyme fusions to phage scaffolds. The stoichiometric ratios for Aml–Hoc, Mal–Hoc, and GK–Hoc are 1:1:1, 1:4:1, and 1:16:1 as indicated. The no enzyme lane contains only the ΔHoc phage, the no phage lane contains only Hoc–enzyme fusions (1:16:1), and wild-type (WT) phage is also shown. Protein bands were visualized by staining gels with GelCode blue safe protein dye.

Wild-type (WT) T4 capsids are composed of mainly gp23, T4 capsid protein, which forms an array of hexamers, and two accessory outer capsid proteins (Hoc and Soc) as indicated in Figure 1A along with the minor capsid protein and portal protein (not shown in Figure 1A; Fokine et al., 2004). Although proteins can be displayed onto the capsids through both Hoc and Soc fusions, we choose Hoc for this work, as there is more intermolecular space for enzyme fusions immobilized onto phage capsids. Future studies could also examine Soc–enzyme fusions to evaluate what effects on the enzymatic activity are obtained when the intermolecular spacing of the fusion enzymes is lessened.

The enzyme–SC and St–Hoc were covalently attached *in vivo* to produce enzyme–SC–St–Hoc (a.k.a. enzyme–Hoc) fusions with a 6 × His tag by co-expressing enzyme–SC and St–Hoc in the same *E. coli* host. The fusion was purified via Ni-Sepharose resins, and separated from un-fused enzyme–SC through size exclusion chromatography (Goldman et al., 2017). Larger enzyme–Hoc fusions eluted earlier than smaller enzyme–SC fusions from the size-exclusion column (Figure S2). This purified material migrated slower on SDS-PAGE gel since the enzyme–Hoc was 40 kDa larger than enzyme–SC (Figure S3). Tailless ΔHoc T4 capsids were produced from ΔHoc T4 phage with a yield of 1–3 mg/L in the presence of 9-aminoacridine, which blocks genomic DNA packaged into the capsids and thus results in no tail attachment to the capsids (Schaerli and Kellenberger, 1980). They show faster electrophoretic mobility than WT T4 capsids as indicated in Figure 1C (lanes 4 vs. 6). The addition of Mal–Hoc onto ΔHoc T4 capsids at various molar ratios slows down their mobility and the Mal–Hoc assembled T4 capsids at ratios over 60–1 (Mal–Hoc: capsid) all maintain the same mobility (Figure 1B), suggesting that the numbers of Mal–Hoc assembled on the phage capsids reached maximum between 60 and 120 (~90 in average; Lanes 4 and 5 in Figure 1B). A darker band with the same position of free Mal–Hoc appears in the ratio of 120–1 (Lane 5 in Figure 1B), indicating that free Mal–Hoc exists following the binding reaction, while there is much less free Mal–Hoc in the ratio of 60–1 in Figure 1B. Likewise, the samples with the ratio of 60–1 for the total enzymes to phage capsids showed the same effect on the electrophoretic mobility as indicated in Figure 1C. Based on these results, we constructed the enzyme assembly onto phage scaffolds at ratios of 40–1 for Maltase alone, while 60–1 was used for the three enzyme mixtures in **Tables 2**, **3** to ensure better basal *K*_cat_ from free enzyme–Hoc fusions for comparison with the assembled ones. Both ratios exhibited minimal background free enzyme–Hoc activity.

### Higher Initial Rate for the Immobilized Mal–Hoc on Phage Capsids

Since Maltase is the rate-limiting step in the enzyme cascade, we measured the initial rate of Mal–Hoc (Klein et al., 2019). The enzyme activity of immobilized Mal–Hoc vs. free Mal–Hoc in solution was measured using 4-Nitrophenyl-α-D-glucopyranoside as a substrate and the yield of the end product of 4-Nitrophenol (4-NP) was monitored by measuring its absorbance at 405 nm. The assembly of Mal–Hoc and phage capsids was conducted at 22°C for 16 h before measuring Mal activity. After prolonged incubation, the immobilized Mal–Hoc on phage capsids still retains its activity and exhibit fourfold higher initial rate relative to the free Mal–Hoc in solution (Figure S4 and Table 1).

**Table 1:** The initial rate for Mal–Hoc fusion with phage scaffold.

**Δ Hoc T4 capsids (nM)**	**Mal–Hoc (nM)**	Initial rate (nM/s)^*^
0	1.80	(4.40 ± 1.12) × 10^2^
0.0450	1.80	(1.73 ± 0.16) × 10^3^
0	0.90	(4.66 ± 0.50) × 10^2^
0.0225	0.90	(1.120 ± 0.16) × 10^3^
0	0.45	(5.54 ± 0.77) × 10^2^
0.0113	0.45	(8.66 ± 1.09) × 10^2^

* *Error bars represent standard deviation (STDEV) derived from technical triplicates*.

### Kinetic Analysis for Enzyme–Hoc Fusions vs. Unfused Enzymes

The kinetics for the three enzyme–Hoc fusions and the three unfused enzymes was measured at a mixing ratio of 1:1:1 of the three enzymes with the total concentration of 0.06 μM. The *V*_max_ was slightly higher for enzyme–Hoc, while *K*_m_ and *K*_cat_ were smaller than enzyme alone (Table 2). Although the *K*_cat_ was slower in enzyme–Hoc, a lower concentration of substrate was needed for enzyme–Hoc to reach 1/2 *V*_max_, resulting in similar catalytic efficiency (*K*_cat_/*K*_m_) for unfused enzymes.

**Table 2:** Kinetics measurement for three enzyme–Hoc fusions vs. non-fusions.

**Enzymes**	**^#^Ratio (Aml:Mal:GK)**	***V*_**max**_ (nM/s)**	***K*_**m**_ (nM)**	^*^K_**cat**_ (1/s)	***K*_**cat**_/*K*_**m**_ (1/M s)**
Hoc–enzyme fusions	1:1:1	6.59 ± 1.56	(5.88 ± 3.31) × 10^4^	0.10 ± 0.03	(1.77 ± 0.79) × 10^3^
Enzyme alone	1:1:1	4.33 ± 1.21	(3.77 ± 1.59) × 10^5^	0.87 ± 0.24	(2.30 ± 1.52) × 10^3^

# *The total concentration is 60 nM*.

* *The number of NAD^+^ converted to NADH per second by the three-enzyme cascade. Error bars are STDEV derived from technical triplicates*.

### Kinetic Analysis for Immobilized Enzyme–Hoc vs. Enzyme–Hoc Free in Solution

The catalysis of a three-enzyme cascade immobilized vs. the mixture of three enzyme fusions free in solution was measured and compared. The immobilized enzymes on phage scaffolds consistently showed higher *K*_cat_ (Table 3) with more enzymatic efficiency (*K*_cat_/*K*_m_) for the conversion of NAD^+^ to NADH. Specifically, assembled enzyme–Hoc fusions with the mixture of three enzymes at a ratio of 1:1:1 appears to have at least 12-fold higher numbers of conversion of NAD^+^ to NADH relative to corresponding enzyme fusions free in solution (Table 3). Likewise, assembled enzyme–Hoc fusions at a mixing ratio of 1:4:1 without changing the total enzyme–Hoc concentration also exhibit an increase in *K*_cat_ of 18-fold relative to the corresponding ones off the scaffolds in solution. However, the sample with a 1:4:1 molar ratio of Aml:Mal:GK did not show better catalytic efficiency than the one with a 1:1:1 ratio of the three enzymes.

**Table 3:** Kinetic measurements for three enzyme–Hoc fusions with phage scaffolds.

**ΔHocT4 capsids (nM)**	**^#^Ratio (A–Hoc:M–Hoc:GK–Hoc)**	***V*_**max**_ (nM/s)**	***K*_**m**_ (nM)**	^*^K_**cat**_ (1/s)	***K*_**cat**_/*K*_**m**_ (1/M s)**
0	1:1:1	6.31 ± 0.12	(3.00 ± 0.71) × 10^5^	0.32 ± 0.01	(1.07 ± 0.14) × 10^3^
1.00	1:1:1	78.50 ± 4.9	(2.50 ± 0.20) × 10^6^	3.93 ± 0.25	(1.57 ± 1.25) × 10^3^
0	1:4:1	9.40 ± 2.04	(1.61 ± 0.59) × 10^5^	0.24 ± 0.05	(1.63 ± 0.91) × 10^3^
1.00	1:4:1	170.00 ± 32.50	(2.31 ± 0.23) × 10^6^	4.26 ± 0.81	(1.83 ± 0.17) × 10^3^

# *The total concentration is 60 nm*.

* *The number of NAD^+^ converted to NADH per second by the three-enzyme cascade. Error bars are STDEV derived from biological replicates*.

## Discussion

### A Naturally Occurring Phage Scaffold for Assembly of Spatially Organized Enzymes

Reconstructed T4 capsid from cryo-electron microscopy (EM) images revealed that Hoc was dumbbell-shaped with a distance, 14 nm, apart from each other, while Soc was rod-shaped closely associated with each other with a few nanometers distance apart (Fokine et al., 2004). Therefore, the distance between two enzyme–Hoc fusions is within the range where substrate channeling can occur between two co-immobilized enzymes within a cascade (Fu et al., 2014; Lim et al., 2019). Consistent with this prediction, our results showed that the immobilized enzyme–Hoc fusions enhanced catalytic activity in comparison to enzymes free in bulk solution. The assembly of Hoc onto phage capsid is mainly driven by the interaction of its C-terminus with T4 capsid protein through electrostatic, Van der Waals, hydrogen bond forces (Sathaliyawala et al., 2010). According to the cryo-EM model, there are ~155 positions for Hoc–enzyme fusions on each ΔHoc capsid; however, the decorated numbers on the capsids depend on the size or charges of the Hoc fusions (Fokine et al., 2004). The addition of enzyme–Hoc fusions significantly changes the electrophoretic mobility of the assembled phage scaffolds, shifting to the same position as WT T4 (lanes 4 and 6 in Figure 1C). Our previous study showed that the loss of Hoc on the capsids (ΔHoc T4) resulted in a lower pI and more negative zeta potential, but no significant change in size (Robertson et al., 2012). Consistently, our results show that ΔHoc T4 migrated faster toward the positive electrode. Based on the estimate from the band density, ~90 copies on average of enzyme–Hoc were decorated on the capsid; therefore, every immobilized enzyme should have at least two neighboring enzymes on the capsids. Having neighboring enzymes likely facilitates the catalysis via substrate channeling. Enhanced stability could also contribute to higher catalytic activity. Thus, we have demonstrated that T4 phage capsids provide a naturally occurring scaffold to immobilize enzymes for efficient biocatalysis.

### Comparable Enzymatic Activity of Enzyme–Hoc Fusion vs. Enzyme Alone

St and SC are two partners of the same domain of *Streptococcus pyogenes* fibronectin-binding protein (FbaB) and the pair is covalently attached through spontaneous amide bond formation between Asp and Lys in the respective partner (Zakeri et al., 2012; Reddington and Howarth, 2015). We individually fused St onto the N-terminus of Hoc and SC to the C-terminus of enzyme to produce enzyme–SC–St–Hoc fusions *in vivo* for subsequent one-step purification. The advantage of St–SC pairing allows the separate expression of enzyme and Hoc in the host and avoids the direct expression of large Hoc fusions, which often have the propensity for improper folding and low yields during the production. By this method, we were able to obtain reasonable yields (~5–10 mg/L) for the enzyme–Hoc fusions with the enzymatic activity comparable to enzymes alone.

### Demonstration of Enhanced Activity for Immobilized Single Enzyme and the Three-Enzyme Cascade

The kinetics were measured after 2 h of assembly and the measurements were finished within 4 h to ensure that the majority of the enzyme fusions were still active. Although the specific molecular mechanism for the three-enzyme cascade is not clearly identified, we attribute the enhancement of the immobilized enzymes mainly to substrate channeling (Miles et al., 1999; Klein et al., 2019). Mal is the rate-limiting step of the three-enzyme cascade (Klein et al., 2019); therefore, we also specifically measured the initial rate for immobilized Mal–Hoc after 16 h of assembly. Our results show that immobilization on phage scaffolds stabilizes the Mal–Hoc after prolonged incubation (Figure S4). Therefore, the enhancement of the immobilized enzyme activity on phage scaffolds is the sum of substrate channeling and improved stability. Although the scaffolded enzyme–Hoc fusions have higher *K*_m_ with reduced binding affinity possibly due to the interference of enzyme active sites while attaching to the scaffolds, the greater benefit of substrate channeling compensates for the disadvantage and results in a higher conversion rate of NAD^+^ to NADH.

In our experiments, the three enzyme–Hoc constructs were incorporated into ΔHoc T4 *in vitro*. This type of *in vitro* synthetic biology has been exploited to circumvent drawbacks to cell-based approaches, such as toxicity and the presence of competing pathways (Koch et al., 2018; Sperl and Sieber, 2018). In addition, *in vitro* assembly allows better control over the stoichiometry of the enzymes in the multi-enzyme-scaffolded complex. However, the T4 system could also be adapted for *in vivo* display of scaffolded enzymes.

## Conclusion

T4 phage scaffolds are composed of prolated protein shells containing arrays of hexamers of capsid protein decorated with Hoc at the center of the hexamers. In this work, we prepared enzyme–Hoc fusions through St–SC pairing and successfully immobilized a three-enzyme cascade, Aml, Mal, and GK, onto naturally occurring T4 phage scaffolds. The immobilized enzymes exhibit enhanced catalytic activity up to 18-fold relative to enzymes free in solution. Thus, we have successfully demonstrated an icosahedral T4 phage scaffold as a new platform for enzyme assembly to enhance biocatalysis applicable to variety of biotechnology applications.

## Data Availability Statement

All datasets generated for this study are included in the article/Supplementary Material.

## Author Contributions

JL designed and conducted the experiments and wrote the manuscript. DZ, JB, and GA performed the experiments and edited the manuscript. EG designed the experiments and wrote the manuscript All authors contributed to the article and approved the submitted version.

## Conflict of Interest

The authors declare that the research was conducted in the absence of any commercial or financial relationships that could be construed as a potential conflict of interest.
