# Supplementary figures and images for: Multi-Enzyme Assembly on T4 Phage Scaffold
Source: Front Bioeng Biotechnol. 2020 Jun 24;8:571. doi: 10.3389/fbioe.2020.00571 (PMC7327620; doi:10.3389/fbioe.2020.00571)

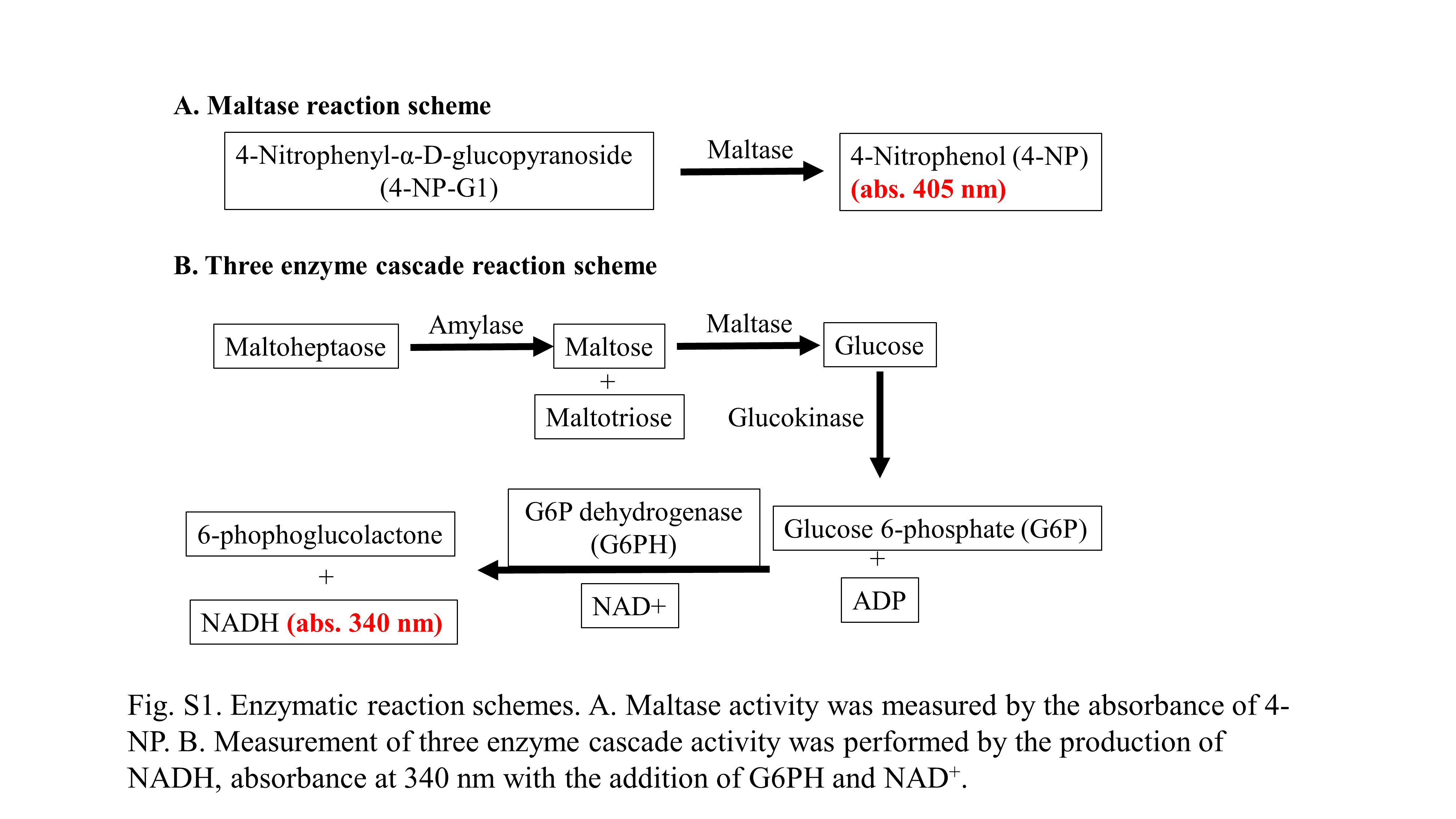

Supplement: Supplementary file 1 [file Image_1.TIF]

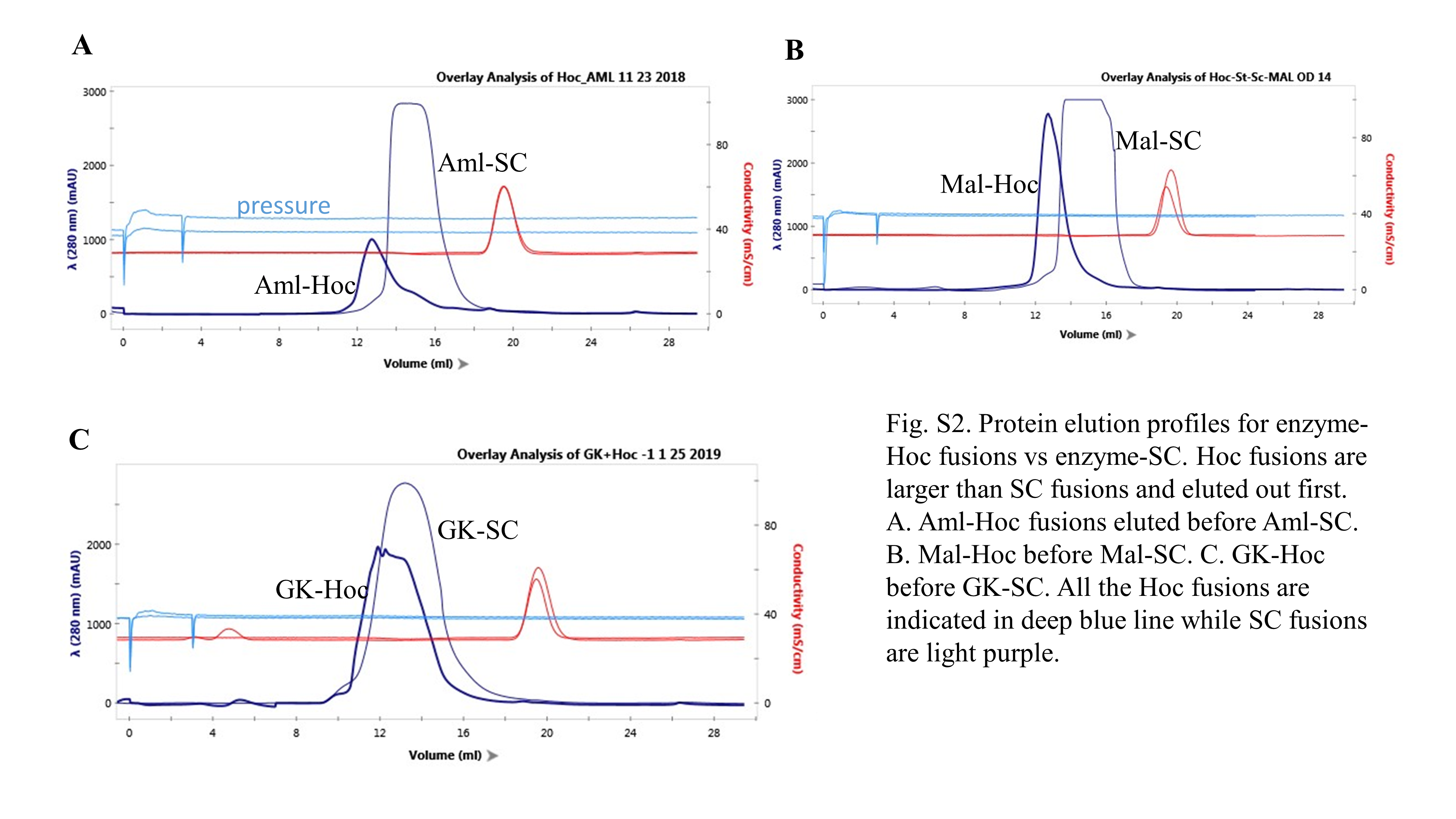

Supplement: Supplementary file 2 [file Image_2.TIF]

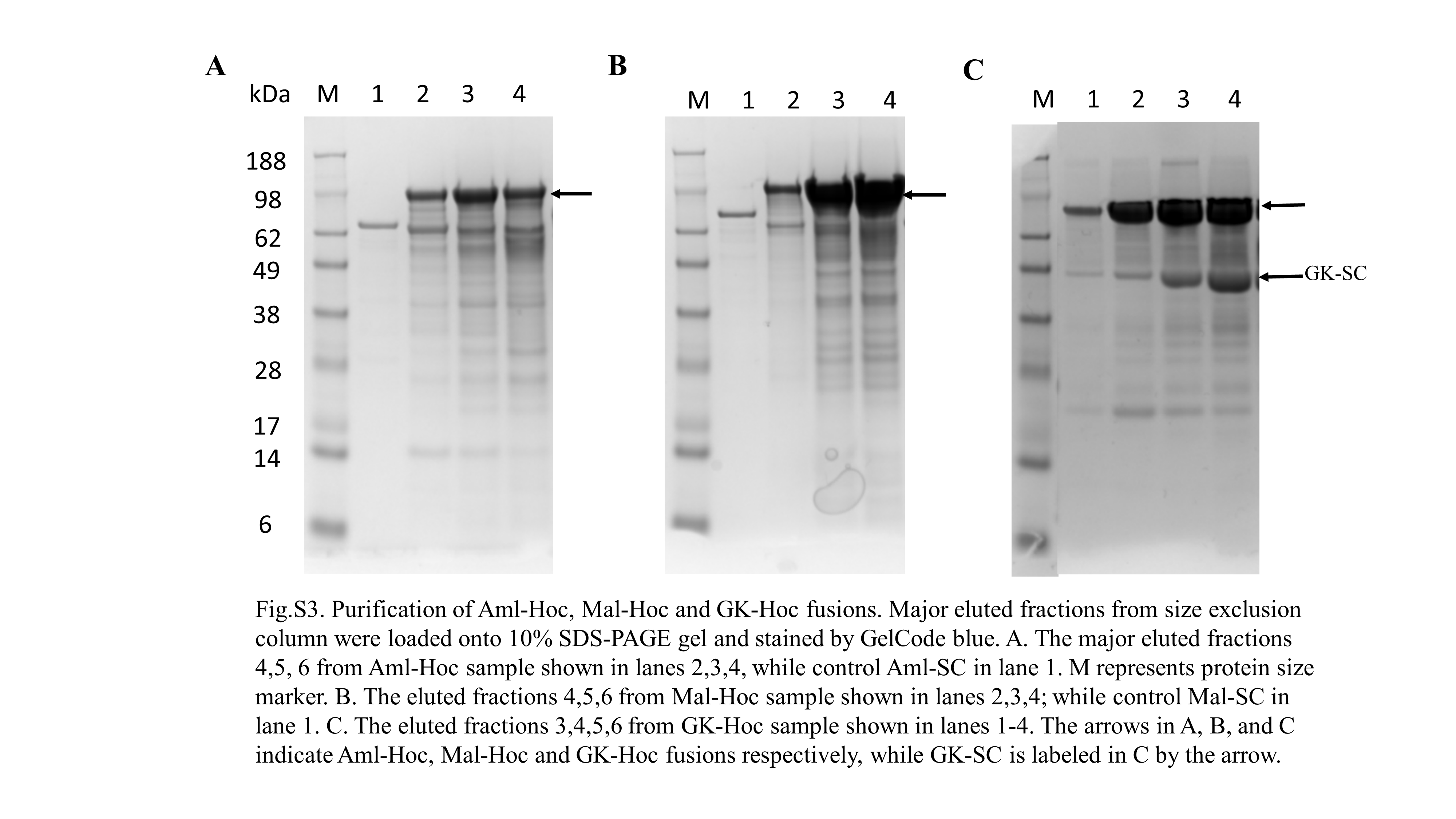

Supplement: Supplementary file 3 [file Image_3.TIF]

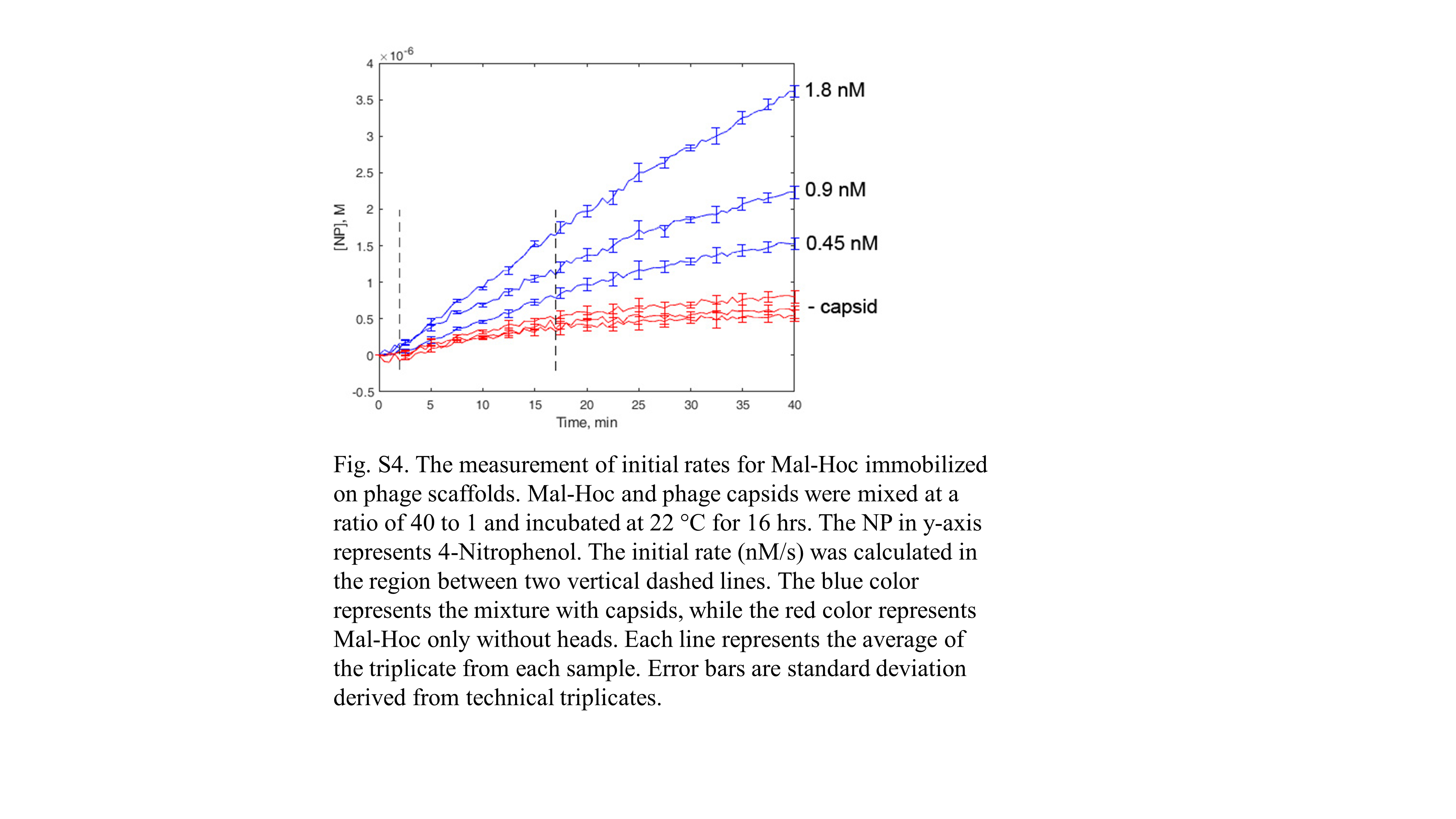

Supplement: Supplementary file 4 [file Image_4.TIF]
