# Supplementary material for: Multi-Enzyme Assembly on T4 Phage Scaffold
Source: Front Bioeng Biotechnol. 2020 Jun 24;8:571. doi: 10.3389/fbioe.2020.00571 (PMC7327620; doi:10.3389/fbioe.2020.00571)
Supplement: Supplementary file 5 [file Data_Sheet_1.pdf]

Supplementary information

A. Primer sequences

Table S1. Primer sequences for cloning enzymes into pET28b and Hoc into pACYCduet.		
Name	Sequence (5'-3')	Cloning vector
*Amylase F	CCTTTT ACCATGGGCGCGGTGAATGG	pET28b
Amylase R	CCTTTTGGATCCAGAACCGCCACCGCCGCTGCCACCCCC TCCCCATGAAGAAGTATGG	pET28b
Maltase F	CCTTTTCCATGGGCATGACCATCTCTGACCAC	pET28b
Maltase R	CCTTTTGGATCCAGAACCGCCACCGCCGCTGCCACCCCC TCCTTTAACCAGGTAGATACG	pET28b
Glucokinase F	GGAATTCCATGGCCGAATTCGCCAGATCTATGACAAAG TATGCATTAGTCG	pET28b
Glucokinase R	AGCGGTCTCGAGGCCGGATCCAGAACCGCCACCGCCGC TGCCACCCCCTCCCAGAATGTGACCTAAGGTCTGG	pET28b
#St-Hoc F1	AAATATCCATGGGCGCGCATATTGTGATGGTGGATGCG TATAAACCGACCAAAATGACTTTTACAGTTGATATAACT CC	pACYC duet
#Hoc Not Xho R1	TGTGTCTCGAGAGTGCGGCCGCCTTATGGATAG1. GTATAGATGATAC	pACYC duet
*The amplified fragments, Aml, Mal and GK using three primer sets, were digested with NcoI and BamHI and then inserted into the compatible sites into our existing pET28-sdAb in BglII format (Goldman, Broussard 2017). # The amplisfied Hoc gene fragement using these two primers were digested with NcoI and NotI and inserted into pACYCduet with the compatible ends.		

B. Gene maps

1.Spytag(St)-Hoc

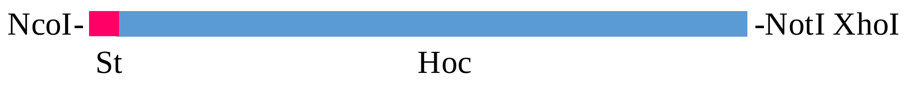

2. Enzymes (Amylase, Maltase, and Glucokinase)

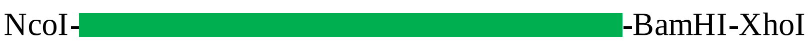

3. Spycatcher

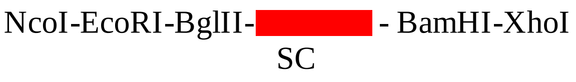

4. Enzyme-SpyCatcher (SC)

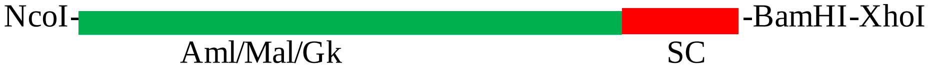

C. Gene sequences

>St-Hoc in pACYCduet
ATGGGCGCGCATATTGTGATGGTGGATGCGTATAAACCGACCAAA**ATGACTTTTAC**
AGTTGATATAACTCCTAAAACACCTACAGGGGTTATTGATGAAACTAAGCAGTTTA
CTGCTACACCCAGTGGTCAAACTGGAGGCGGAACTATTACATATGCTTGGAGCGTAG
ATAATGTTCCACAAGATGGAGCTGAAGCAACTTTTAGTTATGTACTAAAAGGACCTG
CCGGTCAAAAGACTATTAAAGTAGTTGCAACAAATACACTTTCTGAAGGAGGCCCG
GAAACGGCTGAAGCGACAACAACTATCACAGTTAAAAATAAGACACAGACGACTAC
CTTAGCCGTAACTCCTGCTAGTCCCGCGGCTGGAGTGATTGGAACCCCAGTTCAATT
TACTGCTGCCTTAGCTTCTCAACCTGATGGAGCATCTGCTACGTATCAGTGGTATGTA
GATGATTCACAAGTTGGTGGAGAAACTAACTCTACATTTAGCTATACTCCAACTACA
AGTGGAGTAAAAAGAATTAAATGCGTAGCCCAAGTAACCGCGACAGATTATGATGC
ACTAAGCGTTACTTCTAATGAAGTATCATTAACGGTTAATAAGAAGACAATGAATCC
ACAGGTTACATTGACTCCTCCTTCTATTAATGTTCAGCAAGATGCTTCGGCTACATTT
ACGGCTAATGTTACGGGTGCTCCAGAAGAAGCACAAATTACTTACTCATGGAAGAA
AGATTCTTCTCCTGTAGAAGGGTCAACTAACGTATATACTGTCGATACCTCATCTGTT
GGAAGTCAAACTATTGAAGTTACTGCAACTGTTACTGCTGCAGATTATAACCCTGTA
ACCGTTACCAAAACTGGTAATGTAACAGTCACGGCTAAAGTTGCTCCAGAACCAGA
AGGTGAATTACCTTATGTTCATCCTCTTCCACACCGTAGCTCAGCTTACATCTGGTGC
GGTTGGTGGGTTATGGATGAAATCCAAAAAATGACCGAAGAAGGTAAAGATTGGAA
AACTGACGACCCAGATAGTAAATATTACCTGCATCGTTACACTCTCCAGAAGATGAT
GAAAGACTATCCAGAAGTTGATGTCCAAGAATCGCGTAATGGATACATCATTCATA
AAACTGCTTTAGAAACTGGTATCATCTATACCTATCCATAA

>Amylase-SC in pET28b
ACCATGGGCGCGGTGAATGGGAAAGGGATGAATCCAGATTATAAAGCGTATTTAAT
GGCACCGCTCAAAAAAATCCCGGAAGTAACCAACTGGGAGACCTTCGAAAACGACC
TGCGCTGGGCGAAACAGAATGGTTTTTATGCCATTACTGTAGATTTTTGGTGGGGGG
ACATGGAAAAAAACGGGGACCAACAATTCGATTTCTCTTACGCTCAACGCTTTGCTC
AGAGCGTTAAGAACGCAGGTATGAAAATGATCCCGATCATTAGCACCCACCAGTGC
GGTGGTAACGTAGGTGACGATTGCAATGTGCCAATTCCGAGCTGGGTATGGAATCA
GAAATCTGATGATAGCTTATACTTCAAATCGGAGACAGGTACCGTCAATAAAGAAA
CCCTGAATCCGCTGGCGAGTGATGTAATTCGTAAAGAGTATGGCGAATTGTATACCG
CTTTCGCAGCCGCAATGAAACCGTATAAAGATGTTATTGCAAAGATTTATCTCTCCG
GCGGACCAGCTGGTGAGCTGCGTTACCCAAGCTACACGACCAGTGACGGGACGGGT
TATCCAAGCCGCGGCAAGTTTCAGGCGTACACAGAATTTGCAAAATCAAAATTCCGT
TTATGGGTTCTGAACAAATACGGAAGCCTGAACGAAGTCAATAAGGCGTGGGGAAC
CAAACTGATTTCAGAACTGGCTATCTTACCGCCGAGCGATGGCGAACAGTTCCTTAT
GAATGGTTATCTGAGCATGTATGGCAAAGATTATTTGGAATGGTACCAAGGCATCCT

GGAGAATCACACCAAGCTCATCGGGGAACTGGCTCATAATGCTTTTGACACTACGTT
CCAAGTACCGATCGGTGCGAAGATTGCGGGCGTCCATTGGCAATACAACAATCCAA
CGATCCCGCACGGGGCGGAAAAACCGGCTGGNTACAACGATTACAGCCATNTCCTG
GACGCATTCAAATCTGCTAAATTGGACGTCACGTTCACCTGTCTGGAAATGACCGAT
AAAGGTAGCTATCCAGAGTACTCCATGCCGAAAACCTTGGTGCAGAACATCGCCAC
ACTGGCGAACGAGAAGGGGATCGTTTTGAATGGTGAAAATGCGCTCTCTATTGGTA
ATGAGGAGGAATACAAGCGTGTGGCGGAGATGGCATTTAACTATAACTTTGCAGGC
TTCACCCTGCTGCGTTATCAGGACGTTATGTACAATAACTCTCTCATGGGAAAATTT
AAAGATCTGCTGGGCGTCACCCCGGTGATGCAGACGATTGTTGTTAAGAACGTACCA
ACTACCATCGGGGACACAGTTTATATCACAGGGAATCGTGCGGAGCTCGGGTCCTG
GGATACCAAACAGTATCCGATTCAGCTGTACTATGATAGCCACTCAAACGACTGGCG
CGGTAATGTGGTTCTGCCGGCAGAACGCAATATTGAGTTTAAGGCCTTTATTAAATC
AAAAGACGGTACGGTTAAGAGCTGGCAGACTATCCAACAATCCTGGAACCCGGTTC
CTCTCAAAACCACATCCCATACTTCTTCATGG**GGAGGGGGTGGCAGCGGCGGTGG**
CGGTTCTGGATCTGTTGATACCTTATCAGGTTTATCAAGTGAGCAAGGTCAGTCCG
GTGATATGACAATTGAAGAAGATAGTGCTACCCATATTAAATTCTCAAAACGTGATG
AGGACGGCAAAGAGTTAGCTGGTGCAACTATGGAGTTGCGTGATTCATCTGGTAAA
ACTATTAGTACATGGATTTCAGATGGACAAGTGAAAGATTTCTACCTGTATCCAGGA
AAATATACATTTGTCGAAACCGCAGCACCAGACGGTTATGAGGTAGCAACTGCTATT
ACCTTTACAGTTAATGAGCAAGGTCAGGTTACTGTAAATGGCAAAGCAACTAAAGG
TGACGCTCATATTGGATCCGGCCTCGAGCACCACCACCACCACCACTGA

>Maltase-SC in pET28b
ACCATGGGCATGACCATCTCTGACCACCCGGAAACGGAGCCAAAATGGTGGAAAGA
AGCCACCATCTACCAGATTTACCCGGCGAGCTTCAAGGATTCTAATAACGATGGTTG
GGGTGATCTGAAAGGCATTACCTCCAAACTGCAGTACATCAAAGATCTGGGTGTTGA
TGCTATTTGGGTATGCCCGTTCTACGACAGCCCGCAGCAGGACATGGGCTACGATAT
TTCTAACTATGAAAAAGTATGGCCGACCTATGGTACCAACGAGGACTGCTTCGAGCT
GATCGATAAAACGCACAAACTGGGCATGAAATTTATCACGGATCTGGTTATTAACCA
CTGCTCTACTGAACACGAGTGGTTCAAAGAATCTCGCTCTAGCAAAACGAACCCGA
AACGTGACTGGTTCTTTTGGCGCCCGCCTAAAGGTTACGACGCGGAAGGTAAACCG
ATTCCTCCTAACAACTGGAAAAGCTTCTTCGGTGGTTCCGCTTGGACCTTTGACGAA
ACGACGAACGAATTCTACCTGCGTCTGTTCGCCTCTCGTCAGGTTGACCTGAACTGG
GAGAACGAAGACTGTCGTCGTGCGATCTTTGAATCCGCGGTTGGCTTCTGGCTGGAT
CACGGCGTAGATGGCTTCCGCATTGATACGGCAGGTCTGTATTCCAAACGCCCGGGC
CTGCCGGACTCTCCGATCTTCGACAAAACCTCCAAACTGCAGCACCCAAACTGGGGT
TCCCACAACGGCCCGCGTATCCACGAGTATCATCAGGAACTGCACCGCTTCATGAAA
AACCGCGTTAAAGACGGCCGTGAAATCATGACTGTCGGTGAAGTAGCTCACGGTTCT
GACAACGCTCTGTACACTTCTGCGGCTCGTTATGAGGTTAGCGAGGTATTTTCCTTCA
CCCACGTTGAACTGGGCACCTCTCCGTTTTTCCGTTATAACATCGTACCGTTTACGCT
GAAGCAGTGGAAAGAGGCAATCGCATCTAACTTCCTGTTCATCAACGGTACTGACTC
CTGGGCAACGACCTATATCGAGAACCATGATCAGGCTCGCTCTATCACTCGTTTCGC
AGACGATTCCCCGAAGTATCGTAAAATTTCTGGTAAACTGCTGACTCTGCTGGAATG
CTCCCTGACCGGCACCCTGTATGTGTATCAGGGTCAAGAGATCGGTCAAATTAACTT
TAAAGAATGGCCGATCGAAAAATACGAAGACGTTGACGTTAAAAACAACTACGAGA
TCATCAAAAAGTCCTTCGGTAAAAACTCCAAAGAAATGAAAGACTTTTTCAAAGGT

ATCGCCCTGCTGAGCCGCGATCACTCTCGCACGCCGATGCCGTGGACCAAGGATAA
ACCTAACGCGGGTTTTACCGGCCCGGACGTTAAGCCGTGGTTTTTCCTGAATGAATC
TTTTGAACAGGGCATCAATGTAGAGCAGGAGAGCCGTGATGATGACAGCGTGCTGA
ACTTCTGGAAACGTGCACTGCAGGCCCGCAAAAAGTACAAAGAGCTGATGATTTAC
GGCTATGATTTCCAGTTCATTGACCTGGACTCCGACCAGATCTTCTCTTTCACCAAAG
AATACGAAGATAAAACTCTGTTTGCGGCTCTGAACTTCTCTGGCGAAGAAATTGAGT
TCTCCCTGCCGCGTGAAGGCGCCTCCCTGTCTTTCATCCTGGGTAACTATGATGACA
CTGACGTCAGCAGCCGTGTGCTGAAACCTTGGGAGGGTCGTATCTACCTGGTTAAA**G**
GAGGGGGTGGCAGCGGCGGTGGCGGTTCTGGATCTGTTGATACCTTATCAGGTT
TATCAAGTGAGCAAGGTCAGTCCGGTGATATGACAATTGAAGAAGATAGTGCTACC
CATATTAAATTCTCAAAACGTGATGAGGACGGCAAAGAGTTAGCTGGTGCAACTAT
GGAGTTGCGTGATTCATCTGGTAAAACTATTAGTACATGGATTTCAGATGGACAAGT
GAAAGATTTCTACCTGTATCCAGGAAAATATACATTTGTCGAAACCGCAGCACCAGA
CGGTTATGAGGTAGCAACTGCTATTACCTTTACAGTTAATGAGCAAGGTCAGGTTAC
TGTAAATGGCAAAGCAACTAAAGGTGACGCTCATATTGGATCCGGCCTCGAGCACC
ACCACCACCACCACTGA

>Glucokinase-SC in pET28b
ATGGCCGAATTCGCCAGATCTATGACAAAGTATGCATTAGTCGGTGATGTGGGCGGC
ACCAACGCACGTCTTGCTCTGTGTGATATTGCCAGTGGTGAAATCTCGCAGGCTAAG
ACCTATTCAGGGCTTGATTACCCCAGCCTCGAAGCGGTCATTCGCGTTTATCTTGAA
GAACATAAGGTCGAGGTGAAAGACGGCTGTATTGCCATCGCTTGCCCAATTACCGGT
GACTGGGTGGCGATGACCAACCATACCTGGGCGTTCTCAATTGCCGAAATGAAAAA
GAATCTCGGTTTTAGCCATCTGGAAATTATTAACGATTTTACCGCTGTATCGATGGC
GATCCCGATGCTGAAAAAAGAGCATCTGATTCAGTTTGGTGGCGCAGAACCGGTCG
AAGGTAAGCCTATTGCGGTTTACGGTGCCGGAACGGGGCTTGGGGTTGCGCATCTGG
TCCATGTCGATAAGCGTTGGGTAAGCTTGCCAGGCGAAGGCGGTCACGTTGATTTTG
CGCCGAATAGTGAAGAAGAGGCCATTATCCTCGAAATATTGCGTGCGGAAATTGGT
CATGTTTCGGCGGAGCGCGTGCTTTCTGGCCCTGGGCTGGTGAATTTGTATCGCGCA
ATTGTGAAAGCTGACAACCGCCTGCCAGAAAATCTCAAGCCAAAAGATATTACCGA
ACGCGCGCTGGCTGACAGCTGCACCGATTGCCGCCGCGCATTGTCGCTGTTTTGCGT
CATTATGGGCCGTTTTGGCGGCAATCTGGCGCTCAATCTCGGGACATTTGGCGGCGT
GTTTATTGCGGGCGGTATCGTGCCGCGCTTCCTTGAGTTCTTCAAAGCCTCCGGTTTC
CGTGCCGCATTTGAAGATAAAGGGCGCTTTAAAGAATATGTCCATGATATTCCGGTG
TATCTCATCGTCCATGACAATCCGGGCCTTCTCGGTTCCGGTGCACATTTACGCCAG
ACCTTAGGTCACATTCTG**GGAGGGGGTGGCAGCGGCGGTGGCGGTTCTGGATCT**
GTTGATACCTTATCAGGTTTATCAAGTGAGCAAGGTCAGTCCGGTGATATGACAATT
GAAGAAGATAGTGCTACCCATATTAAATTCTCAAAACGTGATGAGGACGGCAAAGA
GTTAGCTGGTGCAACTATGGAGTTGCGTGATTCATCTGGTAAAACTATTAGTACATG
GATTTCAGATGGACAAGTGAAAGATTTCTACCTGTATCCAGGAAAATATACATTTGT
CGAAACCGCAGCACCAGACGGTTATGAGGTAGCAACTGCTATTACCTTTACAGTTAA
TGAGCAAGGTCAGGTTACTGTAAATGGCAAAGCAACTAAAGGTGACGCTCATATTG
GATCCGGCCTCGAGCACCACCACCACCACCACTGA

>Synthesized SC fragments (NcoI-EcoRI-BglI at 5’ and BamHI-XhoI)

CCATGGGCGAATTCAGATCTGTTGATACCTTATCAGGTTTATCAAGTGAGCAAGGTC
AGTCCGGTGATATGACAATTGAAGAAGATAGTGCTACCCATATTAAATTCTCAAAAC
GTGATGAGGACGGCAAAGAGTTAGCTGGTGCAACTATGGAGTTGCGTGATTCATCT
GGTAAAACTATTAGTACATGGATTTCAGATGGACAAGTGAAAGATTTCTACCTGTAT
CCAGGAAAATATACATTTGTCGAAACCGCAGCACCAGACGGTTATGAGGTAGCAAC
TGCTATTACCTTTACAGTTAATGAGCAAGGTCAGGTTACTGTAAATGGCAAAGCAAC
TAAAGGTGACGCTCATATTGGATCCGGCCTCGAGCACCA

D. Construction of enzyme-SC fusions and St-Hoc plasmids

We used the BglBrick cloning strategy (Anderson, Dueber et al. 2010) to produce the enzyme-SC fusions. First a BglBrick compatible version of the pET28b expression vector was constructed by using the quick change mutagenesis kit (Agilent) to remove the unique BglII site. Each enzyme (Aml, Mal, GK) was amplified by polymerase chain reaction (PCR) to include a 10-amino acid glycine-serine linker on their 3' ends. Resulting enzyme gene fragments were subsequently digested with NcoI and BamHI and inserted into similarly digested pET28b. Next, the resulting enzyme BglBricks pET28b DNA was digested with BamHI and XhoI and ligated with SC fragments flanked with BglII and XhoI to produce the enzyme-SC pET28b fusions (Goldman, Broussard et al. 2017). St-Hoc fragments were amplified using PCR with primers containing sequences encoding St, A H I V M V D A Y K P T K, and NcoI site at 5'end and NotI site at 3'end. The resulting St-Hoc fragments containing NcoI and NotI overhang sequences were then inserted into pACYCduet via the compatible sites. The resulting St-Hoc pACYCduet did not have 6xHis. All clones were verified by sequencing. All of the enzymes for cloning were purchased from New England Biolabs (Ipswich, MA)

E. Protein production and purification:

Both enzyme-SC fusion and St-Hoc plasmids were transfected into *E.coli* cells,Tuner (DE3)(F^{-} *ompT hsdS*$_B$ (r_B^{-} m_B^{-}) *gal dcm lacY1*(DE3), and plated onto LB supplemented with kanamycin and chloramphenicol. Next day, one colony from the plate was inoculated into 3 ml of LB containing both kanamycin and chloramphenicol and grown overnight in a 37 °C shaker. One mL of the overnight culture was then inoculated into 500 mL TB supplemented with both antibiotics and shook for 7 to 8 hrs at 37 °C before switching to 25 °C and adding 0.25 mM IPTG. The induced culture was then shook overnight. Cell pellets were obtained the next day by centrifugation at 10,000 x g for 10 min and resuspended in approximately 30 mL phosphate buffer saline (PBS). One mg of Lysozyme was then added to the cell suspension and shook for 5 min at room temperature (RT). Cells were then subjected to sonicating on ice for 2 min with the output cycle 50% (ThermoFisher Scientific, Waltham, MA). The cell suspension were then spun at 20, 000 x g for 15 min and the supernatant was transferred to a new 50 mL conical tube. Subsequently, PBS equilibrated Nickel resin (GE Healthcare) was added and the mixture was rotated in a cold room for 1-2 hrs before pelleting at 1200 x g for 5 min. The resin was washed once with 1x IMAC buffer (20 mM phosphate buffer [pH 7.4], 0.4 M NaCl, and 0.02 M imidazole) and the protein was then eluted with 0.25 M of Imidazole (except for Malt-SC which was eluted with 0.1M Imidazole) and subsequently purified by fast protein liquid chromatography (FPLC) using Enrich SEC 650/70 column with the flow rate 0.5 mL/min and

0.75 mL/fraction. All the eluted fractions were subjected to SDS-polyacrylamide gel electrophoresis (PAGE) (ThermoFisher Scientific, Waltham, MA) to confirm the sizes of enzyme-Hoc fusions. The confirmed enzyme-Hoc fractions were then aliquoted into 3 tubes and stored at -80 °C.

The protein concentration for each fraction was determined by abs.280 nm/molar extinction coefficient (ε) based on Beer's law ($A_\lambda = \varepsilon cL$, where c=[protein]; L=width of light path). The value of ε for each fusion is determined by the composition of Tryptophan, Tyrosine, and Cysteine amino acid and was calculated via free web tool (https://www.novoprolabs.com/tools/protein-extinction-coefficient-calculation).

F. Preparation of tailless ΔHoc T4 phage

Fifteen mL overnight culture grown from a single colony was inoculated into 300 mL of M9 medium supplemented with 0.5% of glucose, 0.1% Casamino acid 2 mM $MgSO_4$, 0.1 mM $CaCl_2$ and 50 µg/mL Tryptophan and shook for 4 hrs at 37 °C until reaching OD 0.45 at 600 nm. *E.coli* cells were then infected with ΔHocT4 phage at Moi (multiple of infection) 2-5 and superinfect again in 7 min followed by the addition of 9-aminoacridine (9-AA) in 30 min. The cells continued shaking for 0.5 hr and the cell pellet was obtained by centrifuging at 10,000 xg for 10 min and resuspended in SM buffer (10 mM,Tris-HCl, NaCl 100 mM, MgSO4 50 mM, Gelatin 0.01%) pH 7.5 supplemented with 1% of $CHCl_3$, 20 µg/mL DNase I, 20 µg/mL RNase A and 10 µg/mL Lysozyme. The suspension was shaken at 37 °C for 1 hr before subjecting to centrifugation at 20000 xg for 30 min. The supernatant was then filtered through Amicon ultra centrifuge filter with cutoff 100 kDa (MilliporeSigma, Burlington, MA) and washed with PBS 6 times to get rids of small proteins, DNA and RNA fragments and concentrate the supernatant. The resulting supernatant was then purified using Superose 6, 10/300 GL (GE Healthcare Life Sciences, Pittsburgh, PA) running on BioRad NGC medium pressure chromatography system equipped with ChromLab 6.0 software(Bio-Rad, Hercules, CA) with the flow rate 0.5 mL /min and 0.75 ml/fraction. The fraction right after void volume was collected.
